# Which Kidney Transplant Recipients Can Benefit from the Initial Tacrolimus Dose Reduction?

**DOI:** 10.1155/2018/4573452

**Published:** 2018-01-30

**Authors:** Kinga Krzyżowska, Aureliusz Kolonko, Piotr Giza, Jerzy Chudek, Andrzej Więcek

**Affiliations:** ^1^Department of Nephrology, Transplantation and Internal Medicine, Medical University of Silesia, Katowice, Poland; ^2^Department of Pathophysiology, Medical University of Silesia, Katowice, Poland; ^3^Department of Internal Diseases and Oncological Chemotherapy, Medical University of Silesia, Katowice, Poland

## Abstract

**Background:**

Observational data suggest that the fixed initial recommended tacrolimus (Tc) dosing (0.2 mg/kg/day) results in supratherapeutic drug levels in some patients during the early posttransplant period. The aim of the study was to analyze a wide panel of patient-related factors and their interactions which increase the risk for first Tc blood level > 15 ng/ml.

**Materials and Methods:**

We performed a retrospective analysis of 488 consecutive adult kidney transplant recipients who were initially treated with triple immunosuppressive regimen containing tacrolimus twice daily. The analysis included the first assessment of Tc trough blood levels and several demographic, anthropometric, laboratory, and comedication data.

**Results:**

The multiple logistic regression analysis showed that age > 55 years, BMI > 24.6 kg/m^2^, blood hemoglobin concentration > 9.5 g/dl, and the presence of anti-HCV antibodies independently increased the risk for first Tc level > 15 ng/ml. The relative risk (RR) for first tacrolimus level > 15 ng/ml was 1.88 (95% CI 1.35–2.64, *p* < 0.001) for patients with one risk factor and 2.81 (2.02–3.89, *p* < 0.001) for patients with two risk factors.

**Conclusions:**

Initial tacrolimus dose reduction should be considered in older, overweight, or obese kidney transplant recipients and in subjects with anti-HCV antibodies. Moreover, dose reduction of tacrolimus is especially important in patients with coexisting multiple risk factors.

## 1. Introduction

Nowadays, tacrolimus (Tc), the calcineurin inhibitor, is most commonly immunosuppressive drug used in kidney transplantation. The dose requirement to achieve tacrolimus target blood concentrations varies substantially between individual patients [1], as well as in a given patient over time [2]. From the beginning, the recommended initial Tc dose was fixed at 0.2 mg/kg/day (as given in Summary of Product Characteristics). In the US, this starting dose has recently been reduced to 0.1–0.15 mg/kg/day in patients receiving basiliximab induction or treated with mycophenolate mofetil. However, it is worth to note that neither manufacturer's Product Characteristics nor most of different countries guidelines definitely recommend such a low tacrolimus starting dose in kidney transplantation setting.

The substantial variability of oral drug absorption and liver metabolism in combination with its narrow therapeutic index result in the need for careful blood Tc concentration monitoring, and subsequent dose adjustment [3]. The most frequent Tc adverse effects include an increased occurrence of delayed kidney graft function [4], posttransplant diabetes mellitus [5], and the whole spectrum of bacterial, viral, or opportunistic infections. Moreover, an unfavorable influence of high tacrolimus blood concentrations on the graft's vasculature has been also described [6]. A potentially graft-deleterious complication is tacrolimus-induced thrombotic microangiopathy, often seen in patients with high Tc blood level [7]. Hence, an inadequately high Tc dose prescribed during the first few days after transplantation may lead to negative outcomes for grafts and patients. According to the current guidelines, the target Tc blood level during the first few weeks after the transplant should not exceed 15 ng/ml [8].

Early reports have shown that the first posttransplant Tc blood level > 15 ng/ml is more frequently observed in older and overweight kidney graft recipients [9]. The effect of recipients' age was also confirmed by other researchers [10, 11]. Moreover, the latter analysis identified five independent risk factors for being slow metabolizers and therefore requiring lower initial Tc doses: male gender, age > 60 years, body mass index (BMI) ≥ 25 kg/m^2^, hepatitis C virus positivity, and low steroid dose [11]. Of note, liver function tests were not analyzed in this study. On the other hand, several medications frequently used in the early period after kidney transplantation may interfere with Tc metabolism, mostly as inducers or inhibitors of hepatic CYP3A5 activity [12, 13].

The primary objective of the present study was to analyze the potential factors influencing the first posttransplant Tc blood level in a large single-center cohort of kidney transplant recipients. To set our research in the everyday clinical practice, we also included the corresponding blood cell count parameters, liver function tests, and all comedications that may alter Tc metabolism.

## 2. Materials and Methods

The Bioethics Committee of the Medical University of Silesia granted permission for this study. Informed consent was not deemed necessary; the majority of data were analyzed anonymously based on prospective center transplant database. Comedication data were retrieved from medical records.

### 2.1. Study Group

**Figure 1:**
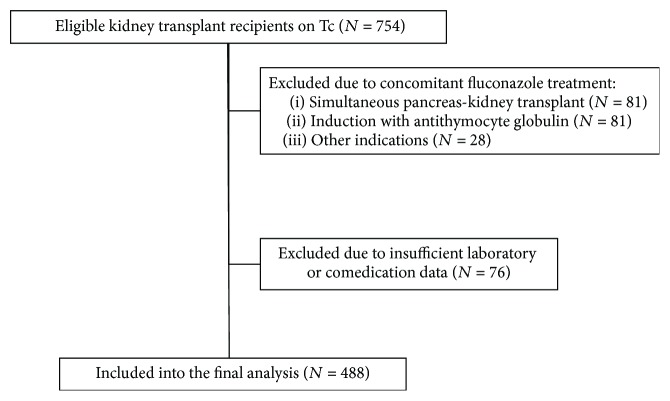
Study flow chart.

Four hundred eighty-eight of 754 consecutive adult kidney graft recipients operated on in our center between 2000 and 2015, who were initially treated with immunosuppressive regimen containing tacrolimus BID, were studied (flow chart, Figure 1). The majority of patients received their grafts from deceased donors (95.7%).

### 2.2. Immunosuppression

After transplantation, most of the patients received triple immunosuppression therapy, which consisted of tacrolimus, mycophenolate or azathioprine or sirolimus, and steroids. The initial Tc dose (in general, 0.2 mg/kg/day) was already given orally prior to the transplantation procedure, and then twice a day, and was administered 2 hours after meal and 1 hour before the next meal. With this recommended dosing we have often observed supratherapeutic blood Tc levels, especially in obese or elderly patients; therefore, since 2011 we started to incidentally reduce the tacrolimus initial dose by up to 20% in such patients, based at the physician's discretion. Besides, a total of 130 patients received basiliximab (Simulect®; Novartis, Basel, Switzerland) as an induction therapy (as those with polyclonal antibodies induction were excluded). Steroids were used in all patients, starting with 500 mg of methylprednisolone (intravenously) during the procedure, 125 mg at first posttransplant day, and then 20 mg of prednisone orally every morning.

In all analyzed patients, the first Tc blood level determination was performed on the morning between the first and fifth postoperative days, at least 24 hours after transplantation procedure. Blood samples for Tc trough level assessments were withdrawn 12 hours after the evening dose. Tc concentrations were assessed using the microparticle enzyme immunoassay (MEIA; Abbott Laboratories, Abbott Park, IL, USA). The upper limit was set at 30 ng/ml, and all values exceeding this result were encoded in the database as 30 ng/ml.

### 2.3. Data Analysis

Nutritional status was scored according to World Health Organization criteria, based on anthropometric measurements performed immediately before the transplantation procedure (underweight: BMI < 18.5 kg/m^2^, normal weight: BMI 18.5–24.99 kg/m^2^, overweight: BMI 25–29.99 kg/m^2^, and obese: BMI ≥ 30 kg/m^2^).

Complete blood count examination was performed on the same day as the initial Tc level measurement; anemia was scored according to World Health Organization criteria. The rest of the biochemical parameters—liver function tests: aspartate aminotransferase (AST), alanine aminotransferase (ALT), and bilirubin—were determined simultaneously or a day later than the initial Tc level measurement.

Initial graft function was defined as immediate (IGF), slow (SGF), or delayed (DGF) graft function. IGF was defined as the serum creatinine concentration (S_Cr_) on the third posttransplant day below or equal to 264 *μ*mol/l (3 mg/dl), SGF was defined as S_Cr_ above 264 *μ*mol/l on the third posttransplant day, and DGF was defined as the need for dialysis therapy during the first week after transplantation.

The diagnosis of new onset diabetes after transplantation (NODAT) was based on WHO criteria, with fasting serum glucose ≥ 126 mg/dL, with or without drug therapy.

Among the medications, used concomitantly and potentially interfering with Tc metabolism, we have analyzed substances which may decrease Tc levels (nitrendipine, proton-pump inhibitors (PPIs), carbamazepine, and trimethoprim-sulfamethoxazole) and those increasing Tc levels (diltiazem, verapamil, H_2_ blockers (mostly ranitidine)). Other potentially interfering comedications (macrolides, HIV antiretroviral therapy, etc.) were not used in the analyzed cohort.

According to current recommendations [8], we have classified the Tc trough blood level > 15 ng/ml as potentially supratherapeutic.

### 2.4. Statistical Analysis

Statistical analyses were performed using STATISTICA 10.0 PL for Windows software package (StatSoft Polska, Kraków, Poland) and MedCalc 12.3.0.0. (Mariakerke, Belgium). Values are presented as means and 95% confidence intervals (CI) or frequencies. The initial comparison was performed for subgroups defined by the first Tc trough level after transplant (≤15 or >15 ng/ml), as well as for the subjects excluded from the analysis. The subgroups were compared using the chi^2^ test (qualitative variables) and analysis of variance (ANOVA; quantitative variables). The univariate analyses included already defined and potential new variables for the risk of supratherapeutic first Tc levels. Correlations were calculated according to Pearson. Multivariate backward regression analysis of factors explaining the variability of first Tc trough blood level (model I) and multiple logistic regression analysis predicting the risk for first Tc trough blood level > 15 ng/ml (model II) in kidney transplant recipients were performed. Both models included age, gender, BMI, pretransplant diabetes, hemoglobin, the presence of anti-HCV antibodies, and Tc initial dose (in mg/kg body weight/day) as potential explanatory variables. Receiver operator curve (ROC) analysis was applied to determine the cut-off values for age, BMI, and hemoglobin level, associated with first Tc blood level > 15 ng/ml. Age and BMI values exceeding the cut-off values were considered risk factors for Tc blood level > 15 ng/ml, and the relative risk (RR) of first Tc blood level > 15 ng/ml was calculated for the subgroups of patients with one or two risk factors present, in relation to the subgroup without them. In addition, we analyzed the percentage of patients with inadequately low (<6 ng/ml) first Tc levels after transplantation and gave their clinical characteristics. In all statistical tests, the *p* values below 0.05 were considered statistically significant; however, for multivariate analyses, a *p* value between 0.05 and 0.1 was interpreted as borderline significant.

## 3. Results

### 3.1. Study Group

The demographic and clinical characteristics of patients included and excluded from the analysis are shown in Table 1. There were 323 men and 165 women in the analyzed group, with 43.2% of overweight or obese and only 9.2% diabetics. In the whole analyzed cohort, there was a small group of anti-HCV positive (with 12 out of 35 treated with interferon-based regimens) and HBs-Ag positive (with 11 out of 16 treated with lamivudine) recipients in the analyzed cohort. Among them, there was no patient with liver cirrhosis or active hepatitis. Increased ALT and AST activity as well as increased bilirubin concentration were present in 51, 18, and 11 recipients, respectively. Increased AST/ALT ratio (>1) was found in 162 subjects. Liver enzyme activities were similar regardless of HBs-Ag and anti-HCV status (data not shown).

The comedications that could potentially interfere with Tc metabolism included PPI (*n* = 354), H_2_-blocker (*n* = 152), nitrendipine (*n* = 79), and trimethoprim-sulfamethoxazole (*n* = 249).

For the entire group, 38.7% of the first measurements exceeded 15 ng/ml. The Tc initial dose was 0.184 mg/kg/day, similar in both study subgroups. The subgroup of patients with first Tc level > 15 ng/ml was significantly older and had higher BMI, greater percentage of pretransplant diabetes, and anti-HCV seropositive subjects. They were also less frequently treated with H_2_-blocker. Notably, the assessment of first Tc trough level was performed slightly earlier in the group with Tc > 15 ng/ml (Table 1).

The subgroup of patients with first Tc level > 15 ng/ml was characterized by insignificantly higher occurrence of early NODAT (24.9 versus 18.7% in patients with first Tc level ≤ 15 ng/ml, *p* = 0.13). Of importance, there were also significantly more opportunistic infections during the first hospital stay (mean 20 ± 10 days) in the higher Tc group (25.4 versus 17.1%, *p* = 0.03).

The systolic blood pressure values at postoperative day (POD) 7, POD 14, and at hospital discharge were similar in both groups (data not shown), whereas the diastolic blood pressure values at the same time-points differed between groups (POD 7: 84.6 versus 86.8 mmHg, *p* = 0.06; POD 14: 80.1 versus 83.5 mmHg, *p* < 0.01, and at discharge: 79.4 versus 81.6 mmHg, *p* = 0.02, with all higher values in the subgroup with first Tc level < 15 ng/ml). Unfortunately, the data regarding the modifications of antihypertensive treatment during the hospital stay are unavailable.

### 3.2. Tacrolimus Dosing and Trough Blood Levels

The first Tc trough blood level was measured on the third day at mean. Notably, the Tc trough blood level and the percentage of levels exceeding 15 ng/ml were not related to the day of assessment. There was also no association between an early graft function and the mean value of first Tc trough level (DGF: 15.1 ± 6.8; SGF: 15.0 ± 7.3; IGF: 14.1 ± 7.0 ng/ml). The highest percentage of first Tc level > 15 ng/ml was noted in DGF subgroup (42.1%), middle percentage in SGF (39.0%), and the lowest in IGF (33.9%); however the trend was not significant (*p* = 0.11). In 6 patients with primary graft non-function (PGN) the mean level was greater (22.0 ± 8.3 ng/ml); however the difference was not significant (*p* = 0.08). Four patients with PGN (67%) had Tc > 15 ng/ml; three out of them were diagnosed with thrombotic microangiopathy.

Tc trough blood level variability was explained by age, gender, nutritional status, the occurrence of diabetes, anemia and anti-HCV antibodies, and the concurrent use of H_2_ blockers (Tables 1 and 2). Additionally, significant correlations were found between the first Tc level and hematocrit values (*r* = 0.234, *p* < 0.001) or hemoglobin concentration (*r* = 0.187, *p* < 0.001). There was no association with liver enzymes activities. More than half of all first Tc levels > 15 ng/ml were observed in the following subsets of recipients: patients > 60 years, overweight and obese, diabetics, and anti-HCV positive subjects.

### 3.3. Pretransplant Risk Factors for First Tacrolimus Trough Blood Level > 15 ng/ml

The multivariate regression analysis (Table 3, model I) revealed that age, BMI, hemoglobin concentration, presence of anti-HCV antibodies, and initial Tc dose/kg/day independently accounted for the variability of the first Tc trough level. Similarly, the multiple logistic regression (model II) showed that age, BMI, hemoglobin concentration, and presence of anti-HCV antibodies were independently increasing the risk for first Tc level > 15 ng/ml. The results of the analysis with hematocrit instead of hemoglobin yielded parallel findings (data not shown).

**Figure 2:**
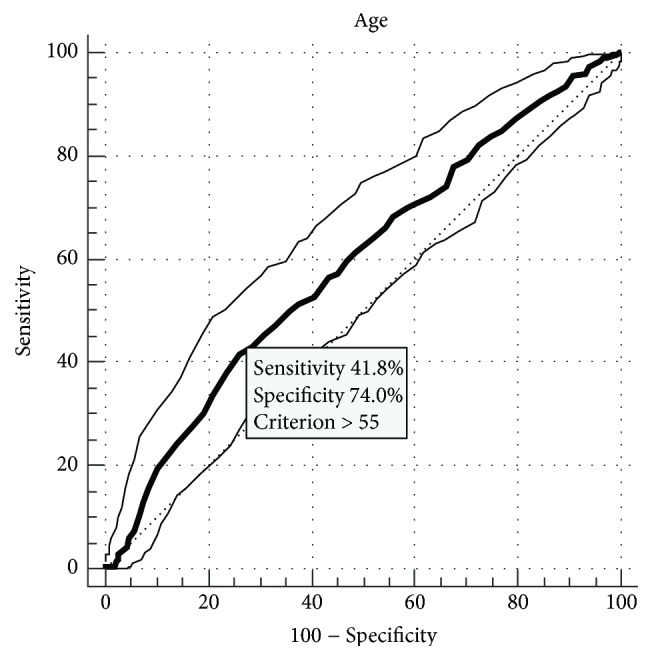
The receiver operator curve analysis for recipient's age which increases the risk for first tacrolimus blood trough level > 15 ng/ml.

The ROC analysis for BMI revealed that values above 24.6 kg/m^2^ increase the risk for first Tc trough blood level > 15 ng/ml with 57% sensitivity and 66% specificity. For age, the cut-off value was 55 years, with 42% sensitivity and 74% specificity (Figure 2). A hemoglobin level below 9.5 g/dl decreased the risk for first Tc trough blood level > 15 ng/ml with 65% sensitivity and 61% specificity.

### 3.4. The Number of Pretransplant Risk Factors and Relative Risk for First Tacrolimus Trough Level > 15 ng/ml

**Figure 3:**
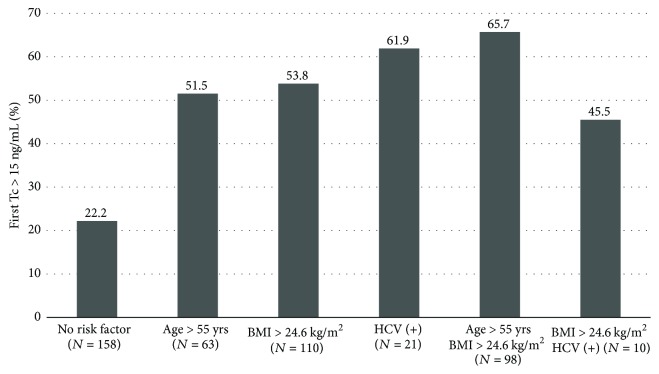
The proportions of patients with first Tc blood trough level > 15 ng/ml in relation to the presence of one or more risk factors, including age > 55 years, BMI > 24.6 kg/m^2^, and presence of anti-HCV antibodies.

We divided the patients into subgroups, based on the number of risk factors known prior to transplantation procedure. Among them, we included recipient's age and BMI values exceeding the cut-off values from ROC analysis, and the presence of anti-HCV antibodies. There were 158 patients without, 194 with one, 111 with two, and only 1 patient with all three risk factors.  Figure 3 shows the proportions of patients with first Tc blood level > 15 ng/ml in relation to the presence of one or more risk factors mentioned above. The RR for first Tc level > 15 ng/ml was 1.88 (95% CI, 1.35–2.64; *p* < 0.001) for patients with one risk factor and 2.81 (95% CI, 2.02–3.89; *p* < 0.001) for patients with two risk factors, in comparison with the subgroup without any risk factors present.

The analysis of early graft function revealed a significant increasing trend for the occurrence of DGF in study subgroups with zero, one, or two above risk factors present (20.3 versus 30.4 versus 31.3% respectively, *p* = 0.03). There was also a reciprocal trend for the IGF frequency across those subgroups (36.1 versus 20.1 versus 15.3% respectively, *p* < 0.001).

Notably, the occurrence of infectious complications in the early posttransplant period was significantly higher in patients with 1 or 2 risk factors as compared with no risk factors subgroup (*p* = 0.03), while the occurrence of posttransplant diabetes was similar in the corresponding analysis (data not shown).

### 3.5. Effects of Initially Reduced Tacrolimus Dosing

Reduced doses of tacrolimus (0.16–0.19 mg/kg/day) were received by 172 patients (35.2%). It resulted in decreased frequency of first Tc levels > 15 ng/ml in patients > 55 years (48.0 versus 56.4%), subjects with BMI > 24.6 kg/m^2^ (52.5 versus 55.0%), and patients with both those risk factors (62.9 versus 69.4%). However, these differences were not statistically significant.

Among patients with reduced Tc dosing (<0.19 mg/kg/day), only 3.4% presented first Tc level below 6 ng/ml (potentially subtherapeutic). Of note, the percentage of patients with first Tc level < 6 ng/ml among standard Tc dose recipients was 5.7%. The subgroup of patients with first Tc level < 6 ng/ml was significantly younger [38 (32–43) versus 48 (46–49), *p* < 0.001], had similar BMI [23.1 (21.5–24.8) versus 24.6 (24.0–24.9), *p* = 0.13], and tended to have lower hemoglobin level [9.0 (8.2–9.7) versus 9.6 (9.4–9.7), *p* = 0.09] than patients with first Tc level ≥ 6 ng/ml.

## 4. Discussion

The primary aim of our study was an in-depth analysis of a wide spectrum of potentially relevant demographic, laboratory, and clinical factors, which may supposedly influence the first postkidney transplant Tc trough level. Except for those previously proposed, we have also analyzed other potential confounders, such as residual diuresis, dialysis vintage, retransplant, presence of HBs antigen, and an early graft function. Additionally, we have analyzed blood cell counts (hemoglobin and hematocrit), as changes in these parameters were shown to influence the whole blood Tc concentration, and liver function tests (including AST/ALT ratio) for a more accurate interpretation of the hypothetical impact of hepatitis B virus and hepatitis C virus status on tacrolimus metabolism. We have also carefully analyzed the concomitant use of all medications, which were described by drug manufacturers as potentially modifying the drug trough concentrations.

In the present study, recipient's age, BMI, hemoglobin level, and the presence of anti-HCV antibodies were shown to be independent risk factors for the potentially toxic first Tc whole blood trough level after kidney transplantation. Aside from blood cell count parameters, which are supposed to change during the perioperative period, the three other confounders are always known at the day of transplantation. Therefore, they should be taken into consideration when calculating the initial Tc dose. The presently obtained cut-off value for age (>55 years) is in line with the recent findings of Størset et al., wherein Tc bioavailability increases with age in both genders and remains relatively constant in patients aged > 55 years [14]. In older patients, despite the decreasing drug absorption, the volume of distribution for lipophilic drugs increases [15]. Concurrently, decrease in liver mass and hepatic blood flow [16], together with reduced CYP3A5 activity [17], may further contribute to the high first Tc blood level. Unfortunately, elderly patients are more prone to Tc toxicity [18]. Nevertheless, there is a lack of recommendations concerning initial Tc dose reduction in elderly kidney transplant recipients, although the targeted troughs in the first posttransplant months are suggested to be lower [15, 18]. Of interest, in our study the variability of first Tc trough level was hardly affected by the range of initial Tc doses covered by this analysis (0.16–0.21 mg/kg/day). As a consequence, the percentage of supratherapeutic as well as subtherapeutic first Tc trough levels in the reduced dosing subgroup was similar to standard dosing subgroup. This supports the supposition that the reduction of initial Tc dose in older and obese kidney transplant recipients was inadequate.

BMI, which generally increases with age, was shown to be independently related to increased Tc first level, when its value exceeded 24.6 kg/m^2^. It is probably partly due to the altered body fat mass in those patients, as it was shown that Tc dose monitoring based on bioimpedance-derived body composition may provide more adequate dosage that based on BMI [19]. Of note, in our study, the highest frequency of subjects with first supratherapeutic Tc blood level was observed in the subgroup where both age and BMI exceeded the cut-off values. Moreover, an increased body mass seems to be a main cause of relatively high occurrence of NODAT, particularly in exceed Tc group.

In the whole analyzed group, there was only a small number of patients (10.5%) with positive HCV serology or presence of HBs antigen, and the vast majority had normal liver function test results. Despite this, the HCV(+) subgroup presented the second highest frequency of patients with first Tc blood level > 15 ng/ml, whereas there was no association between the first Tc level and liver enzymes activity at the time of Tc measurement. Notably, in the HBs (+) subgroup we have observed a similar proportion of patients with supratherapeutic Tc level (50% versus 54.3% in the HCV subgroup); however, the difference between HBs (+) and HBs (−) patients did not reach significance, owing to the probe size. To date, Trotter et al. have noted a lower Tc dose requirement in hepatitis C-infected versus noninfected patients after liver transplantation [20]. The most possible explanation was the decreased hepatic drug clearance caused by mild hepatic injury from recurrent hepatitis C virus [20]. Correspondingly, in cohort of patients with liver transplant, who were treated with anti-HCV interferon-based regimen, responders (with HCV-RNA negative after treatment) presented a 31.8% decline in calcineurin inhibitor trough level, which resulted in a 50% rate of acute cellular graft rejection [21]. This supports the hypothesis that viral eradication improved the liver microsomal metabolic function.

It is worth noting that the presence of above defined risk factors resulted not only in the increased percentage of first supratherapeutic Tc level, but also in the increased occurrence of potentially deleterious clinical complications after kidney transplantation, that is, DGF and infections during the hospital stay.

One of the main limitations of our study regardless of its retrospective character is the method of tacrolimus level assessment, MEIA. That is considered inferior to more specific liquid chromatography with mass spectrometric detection, which is used only by several transplant centers [22]. Another limitation is the lack of CYP3A5 genotyping. Of note, in the homogenic Polish Caucasian population, the vast majority of subjects (approximately 90%) are CYP3A5*∗*3*∗*3 homozygotes [23], being slow calcineurin inhibitor metabolizers. Moreover, to date, CYP3A5 genotyping is neither recommended nor routinely performed prior to transplantation; thus it cannot be used for initial tacrolimus dose calculation in daily clinical practice. It has also been showed recently that the optimization of initial Tc dose using pharmacogenetics testing does not improve clinical outcomes [24]. It should be also acknowledged that in our center the first determination of tacrolimus level was performed at different time-points after transplantation, mostly depending on laboratory constraints. Nevertheless, the Tc trough levels were not related to the day of assessment. Finally, we were not able to analyze the potential influence of positive HBs antigen status on the first Tc level due to the small number of HBs (+) patients.

Nevertheless, our present study has several strengths. Except for the solely age-dependent differences study by Jacobson et al. [10], our analysis contains the largest-to-date population of kidney transplant recipients. Regardless of the fact that we have included all known (to date) clinical risk factors, which may affect Tc metabolism, we have also recorded and analyzed the potentially interfering comedications, as well as corresponding values of blood cell count and liver function tests, which had not been previously analyzed in this setting. Finally, using ROC analysis, we have also calculated the clinically useful cut-off values for the recipient's age and BMI, identifying the subgroup of patients with markedly increased risk of having supratherapeutic Tc level during first few days after kidney transplantation.

In conclusion, initial Tc dose reduction should be considered in kidney transplant recipients aged 55 years or older, who are overweight or obese, and patients with the presence of anti-HCV antibodies. There is also a greater risk of Tc overdose in patients without anemia. Our analysis precludes detailed calculation of the dose reduction needed in patients with demonstrated risk factors. Further prospective studies are warranted with intentional Tc dose reduction in risk-loaded patients.

## Figures and Tables

**Table 1:** Clinical characteristics and laboratory parameters in patients included into the analysis (stratified to the first posttransplant Tc trough level) with comparison to the excluded cohort.

	Excluded *n* = 266	Included *n* = 488	ANOVA or chi^2^	First Tc ≤ 15 ng/ml *n* = 299	First Tc > 15 ng/ml *n* = 189	ANOVA or chi^2^
Age [years]	43 (42–45)	47 (46–49)	<0.001	45 (44–47)	51 (49–53)	<0.001
Gender [M/F]	137/129	323/165	<0.001	187/112	136/53	0.04
BMI [kg/m^2^]	23.4 (22.9–23.8)	24.6 (24.2–24.9)	<0.001	23.6 (23.2–24.1)	25.5 (25.0–26.0)	<0.001
Overweight/obese [%]	22.6/2.3	37.1/6.1	<0.001	28.4/5.4	50.8/7.4	<0.001
Dialysis vintage [mo]	46 (41–51)	39 (36–42)	0.17	39 (35–43)	40 (36–45)	0.66
Residual diuresis [ml]	620 (519–722)	626 (558–693)	0.89	622 (536–709)	612 (505–718)	0.88
Hypertension [%]	89.8	91.0	0.70	91.3	90.5	0.80
Diabetes [%]	34.6	9.2	<0.001	6.7	13.2	0.02
Anti-HCV positive [%]	13.9	7.2	0.004	5.4	10.1	0.07
HBs antigen positive [%]	3.4	3.3	0.89	2.7	4.2	0.50
CIT [h]	16 (15–17)	18 (17–19)	0.008	18 (17–19)	18 (17–19)	0.97
First transplant [%]	75.6	86.2	<0.001	86.0	86.8	0.90
Induction [%]	49.2	26.6	<0.001	26.4	27.0	0.97
H_2_-blocker [%]	15.0	31.1	<0.001	34.8	25.4	0.04
PPIs [%]	81.2	72.5	0.01	71.6	74.1	0.85
Tc initial dose [mg/kg]	0.173 (0.168–0.179)	0.184 (0.181–0.187)	<0.001	0.186 (0.182–0.189)	0.182 (0.178–0.186)	0.22
Tc first trough level [ng/ml]	16.1 (15.1–17.1)	14.7 (14.0–15.3)	0.27	9.9 (9.6–10.2)	22.3 (21.5–23.0)	<0.001
Day of first Tc assessment	1.7 (1.5–1.9)	3.3 (3.2–3.4)	<0.001	3.5 (3.4–3.6)	3.0 (2.8–3.1)	<0.001
Serum creatinine	493 (455–530)	538 (510–565)	0.15	520 (486–555)	564 (519–610)	0.12
Delayed graft function [%]	27.8	26.8	0.84	25.1	29.6	0.32

Data shown as means ± 95% CI or frequencies. *Statistics*: *p values below 0.05 are considered statistically significant*. BMI: body mass index, HCV: hepatitis C virus, HBs: hepatitis B surface antigen, CIT: cold ischemia time, HLA: human leukocyte antigen, H2-blocker: H2 receptor blocker, PPIs: proton-pump inhibitors, and Tc: tacrolimus.

**Table 2:** The mean values of first tacrolimus blood trough level after kidney transplantation (left column) and the proportion of patients with first tacrolimus blood trough level exceeding 15 ng/ml (right column), presented in subgroups of patients with or without the presence of potential risk factor.

	Tc first level [ng/ml]	Tc > 15 ng/ml *n* [%]
Gender		
Men (*n* = 323)	**15.3 (14.5**–**16.1)**^*∗∗*^	**n** ** = 136 (42.1)** ^*∗*^
Women (*n* = 165)	13.4 (12.5–14.4)	*n* = 53 (32.1)

Nutritional status		
Underweight (*n* = 23)	12.2 (9.5–14.8)	*n* = 6 (26.1)
Normal weight (*n* = 247)	12.6 (11.8–13.4)	*n* = 66 (26.7)
Overweight (*n* = 182)	**16.9 (15.9**–**18.0)**^∧∧∧^	**n** ** = 95 (52.2)** ^∧∧∧^
Obesity (*n* = 36)	**18.7 (16.1**–**21.2)**^∧∧∧^	**n** ** = 22 (61.1)** ^∧∧∧^

Diabetes mellitus		
Yes (*n* = 44)	**17.1 (14.8**–**19.4)**^*∗*^	**n** ** = 25 (56.8)** ^*∗*^
No (*n* = 444)	14.4 (13.7–15.1)	*n* = 164 (36.9)

Anti-HCV positive		
Yes (*n* = 35)	**17.0 (14.3–19.6)** ^*∗*^	**n** ** = 19 (54.3)** ^*∗*^
No (*n* = 453)	14.6 (14.0–15.3)	*n* = 170 (37.5)

HBs positive		
Yes (*n* = 16)	15.7 (11.5–20.0)	*n* = 8 (50)
No (*n* = 462)	14.8 (14.1–15.4)	*n* = 173 (37.4)

ALT > 40 IU/L		
Yes (*n* = 51)	14.6 (12.6–16.5)	*n* = 19 (37.3)
No (*n* = 421)	14.7 (14.1–15.4)	*n* = 165 (39.4)

AST > 40 IU/L		
Yes (*n* = 18)	17.5 (13.3–21.7)	*n* = 9 (50.0)
No (*n* = 434)	14.6 (13.9–15.2)	*n* = 168 (38.9)

AST/ALT > 1		
Yes (*n* = 162)	15.1 (14.0–16.2)	*n* = 68 (42.0)
No (*n* = 286)	14.4 (13.6–15.3)	*n* = 108 (37.8)

Anemia		
Severe (*n* = 77)	**12.6 (11.1**–**14.2)**^###^	**n** ** = 21 (27.3)** ^###^
Mild (*n* = 219)	**13.2 (12.4**–**14.1)**^###^	**n** ** = 65 (29.7)** ^###^
No (*n* = 192)	17.1 (16.0–18.2)	*n* = 103 (53.6)

Transplant No		
First (*n* = 421)	14.7 (14.1–15.4)	*n* = 164 (39.0)
Next (*n* = 67)	14.5 (12.7–16.3)	*n* = 25 (37.3)

Early graft function		
IGF (*n* = 121)	14.1 (12.8–15.4)	*n* = 41 (33.9)
SGF (*n* = 228)	15.0 (14.0–16.0)	*n* = 89 (39.0)
DGF (*n* = 137)	15.1 (13.8–16.3)	*n* = 59 (42.1)

H2-blocker		
Yes (*n* = 152)	**13.5 (12.4**–**14.5)**^*∗*^	**n** ** = 48 (31.6)** ^*∗*^
No (*n* = 336)	15.2 (14.4–16.0)	*n* = 141 (42.0)

PPI		
Yes (*n* = 354)	14.9 (14.2–15.6)	*n* = 140 (39.5)
No (*n* = 134)	14.0 (12.8–15.3)	*n* = 49 (36.6)

Data shown as means ± 95% CI or frequencies. BMI: body mass index, HCV: hepatitis C virus, HBs: hepatitis B surface antigen, IGF: immediate graft function, SGF: slow graft function, DGF: delayed graft function. Statistical significance: ^∧∧∧^*p* < 0.001 versus normal weight; ^###^*p* < 0.001 versus no anemia, ^*∗*^*p* < 0.05 or ^*∗∗*^*p* <0.01 versus the second value without the presence of risk factor.

**Table 3:** Multivariate backward regression analysis of factors explaining variability of the first tacrolimus trough level (model I) and multiple logistic regression predicting the risk of first tacrolimus trough level > 15 ng/ml (model II) in kidney transplant recipients. Both models included age, gender, BMI, pretransplant diabetes, hemoglobin, the presence of anti-HCV antibodies, and tacrolimus initial dose in mg/kg of body weight/day.

Independent variable	Model I		Model II	
	*β* ± SD	*p*	OR (95% CI)	*p*
Age [per year]	0.105 ± 0,022	<0.001	1.02 (1.01–1.04)	0.003
BMI [per unit]	0.417 ± 0.082	<0.001	1.10 (1.05–1.16)	<0.001
Hemoglobin [per 1 g/dL]	0.607 ± 0.140	<0.001	1.25 (1.12–1.40)	<0.001
Anti-HCV positive	4.942 ± 1.024	<0.001	3.22 (1.64–6.31)	<0.001
Tacrolimus dose [per 1 mg/kg/day]	16.457 ± 9.321	0.08	-	

SD: standard deviation, OR: odds ratio, BMI: body mass index, and HCV: hepatitis C virus. Statistics: *p* values below 0.05 were considered statistically significant, and *p* value between 0.05 and 0.1 was interpreted as borderline significant.
